# MiRNA-210 is involved in cigarette smoke extract-induced apoptosis of MLE-12 via the Shh signaling pathway

**DOI:** 10.18332/tid/186643

**Published:** 2024-05-29

**Authors:** Zhongshang Dai, Zijie Zhan, Yan Chen, Jinhua Li

**Affiliations:** 1 Department of Infectious Diseases, The Second Xiangya Hospital, Central South University, Changsha, China; 2 Department of Pulmonary and Critical Care Medicine, the Second Xiangya Hospital, Central South University, Changsha, China; 3 Research Unit of Respiratory Disease, Central South University, Changsha, China; 4 Clinical Medical Research Center for Pulmonary and Critical Care Medicine in Hunan Province, Changsha, China; 5 Diagnosis and Treatment Center of Respiratory Disease, Central South University, Changsha, China

**Keywords:** cigarette smoke extract, apoptosis, MiR-210, COPD

## Abstract

**INTRODUCTION:**

The aim of the study is the regulatory effect of MicroRNA-210 (MiR-210) on cigarette smoke extract (CSE)-induced mouse lung epithelial type II cells (MLE-12) apoptosis and determine whether the MiR-210 is involved in cigarette smoke extract-induced apoptosis of MLE-12 via Shh signaling pathway.

**METHODS:**

Expression of MiR-210 in CSE-induced MLE-12 was assessed by qRT-PCR. The emphysema mouse model and MiR-210 knockdown mice were each established by inhaling cigarette smoke or intratracheal lentiviral vector instillation. The Sonic hedgehog (Shh), Ptch1, Gli1, B-cell lymphoma-2 (Bcl-2), and Caspase 3 protein expressions were detected by Western blotting. mRNA expressions of MiR-210, Shh, Ptch1, and Gli1 were measured using quantitative real-time polymerase chain reaction (qRT-PCR). Apoptotic ratios in mice and CSE-induced HPVEC were assessed using TUNEL (terminal deoxynucleotidyl transferase dUTP nick end labeling) assays and flow cytometry.

**RESULTS:**

Our results showed that MiR-210 mRNA levels were significantly down-regulated in the CSE-induced MLE 12. MLE 12 apoptosis with down-regulated Shh, Ptch1, Gli1, and Bcl-2 expression, increased Caspase 3 expression in the emphysema mouse model and CSE-induced MLE 12. Knockdown MiR-210 can facilitate cell apoptosis and emphysema via the Shh signaling pathway in mice. *In vitro*, MiR-210 can attenuate the apoptosis of CSE-exposed MLE 12. Moreover, MiR-210 regulated the Shh pathway and promoted its expression.

**CONCLUSIONS:**

MiRNA-210 is involved in cigarette smoke extract-induced apoptosis of MLE-12 via the Shh signaling pathway. The present study reveals that MiRNA-210 may be a key regulator of cellular apoptosis and could be explored as a potential therapeutic target in the future.

## INTRODUCTION

Chronic obstructive pulmonary disease (COPD) has become a global public health challenge due to its high prevalence and associated mortality^1,2^. The China Pulmonary Health (CPH) study reported that the overall prevalence of spirometry-defined COPD was 8.6% among the general Chinese population aged 20 years and older, with an estimated total of 99.9 million affected individuals^3^. But the pathogenesis remains poorly understood. Cigarette smoking is a well-established risk factor for COPD, and our previous research demonstrated that cigarette smoking accelerates disease progression by inducing apoptosis in bronchial and alveolar epithelial cells^4,5^. Alveolar type II epithelial cells (AT2) are essential for maintaining the distal lung epithelium and for regeneration following injury. Dysfunction of these cells contributes to the development of various parenchymal lung diseases, including emphysema^6^. Cigarette smoke extract (CSE) induces apoptosis in AT2 cells, as well as in endothelial and smooth muscle cells^7,8^. Apoptosis of epithelial cells is strongly associated with the pathogenesis of COPD in vivo and is a major focus of research. Growing evidence indicates that the Sonic hedgehog (Shh) pathway plays a role in stem cell maintenance, likely by promoting stem cell proliferation^9^. The canonical Sonic hedgehog (Shh) pathway begins with Shh secretion and binding to its target cell's transmembrane protein patched (Ptch1). This interaction lifts the repression on smoothened (Smo), a transmembrane protein, leading to the activation of Gli transcription factors (Gli1-3) and ultimately regulating gene transcription^5^. Numerous studies have highlighted the anti-apoptotic effects of the Shh signaling pathway^10-12^. Additionally, recent research has shown that hyperoxia-induced oxidative stress leads to apoptosis in AT2 cells by inhibiting the Shh pathway^13^.

MicroRNAs (MiRNAs) are short transcripts, 19-25 nucleotides in length, that do not code for proteins^14^. They are increasingly recognized for their involvement in numerous biological processes, including chromatin modification, regulation of protein activity, and gene imprinting^15^. Recent studies have demonstrated that exposure to cigarette smoke causes significant changes in MiRNA expression in both humans and rats^16,17^. Devlin et al. identified MicroRNA-210 (MiR-210) as a significant target of the hypoxia-inducible factor, noting its overexpression in various cardiovascular diseases and solid tumors. Elevated levels of MiR-210 are associated with an in vivo hypoxic signature and correlate with poor prognosis in cancer patients^18^. Mateu-Jimenez et al.^19^ showed that expression of MiR-210 and DNA methylation was greater in lung tumor specimens of lung cancer (LC)-COPD than of LC patients. Another study demonstrated cigarette smoke-induced relative up-regulation of cellular and extracellular vesicles (EVs) MiR-210 expression of bronchial epithelial cells by evaluating the modified EVs and COPD lung samples^20^. However, whether MiR-210 has a role in the progress of COPD by regulating the function of AT2 remains unclear. Based on the above studies, we postulated that MiRNA-210 is involved in cigarette smoke extract-induced apoptosis of MLE-12 via the Shh signaling pathway.

## METHODS

### Animals

This animal protocol was approved by the Ethics Committee of the Second Xiangya Hospital of Central South University. Six-week-old male C57BL/6J mice were divided into four groups (n=5 per group). The control group was exposed to normal air from 8 to 16 weeks of age. The CS group was exposed to cigarette smoke (CS) four times a day during the same period^4^. The CS + Vector group received a lentiviral empty vector (10^7^ pfu per mouse, once a week, intratracheally) at 6 and 7 weeks old, then exposed to CS from 8 to 20 weeks old^4^. The CS + Si-MiR-210 group was treated with a lentiviral miR-210 inhibitor (10^7^ pfu per mouse, once a week, intratracheally) at 6 and 7 weeks old. One mouse from the CS + Vector group and one from the CS + Si-MiR-210 group died during the experiment. The left lung tissues of the mice were inflated with 10% formalin at a constant pressure of 25 cm H_2_O for 24 hours, then fixed and embedded.

### Cell lines and culture

Mouse lung epithelial type II cells (MLE-12) were obtained from ATCC and cultured in the recommended medium with 5% fetal bovine serum at 37°C in a humidified atmosphere containing 5% CO_2_. The medium was changed every two days. Before exposure to CSE, shRNA, and/or lentivirus, the cells were starved for 24 hours. MiR-210 and the vector lentivirus were transfected into MLE-12 cells following the manufacturer's guidelines, with a multiplicity of infection of 10. Cells expressing green fluorescent protein were considered successfully infected.

### Preparation of CSE

Half a cigarette (Marlboro, China) was smoked through a 0.22 mm filter to remove particles and bacteria, with the smoke collected into a vessel containing 20 mL of 5% fetal bovine serum. This mixture was the starting solution for CSE, with a pH of 7.4. Fresh CSE was prepared before each experiment and diluted to a 5% working concentration.

### Transient transfection of miR-210 inhibitor

Cells were seeded into six-well plates and allowed to grow overnight until they reached 70-90% confluence. To downregulate miR-210 expression, MLE-12 cells were transfected with specific inhibitors or a miRNA-negative control (Ambion, USA) using Lipofectamine 2000 (Invitrogen, Carlsbad, USA) following the manufacturer's protocol. Experiments were conducted 48 hours after transfection.

### Morphology and apoptosis assessment

Lung tissue samples were fixed in 4% formaldehyde, sectioned into 3.5 mm thick slices, and stained with hematoxylin and eosin (HE). Emphysema was quantified by assessing alveolar destruction, using the mean linear intercept (MLI) and destruction index (DI). MLI was calculated by dividing the length of a line drawn across the section by the total number of intercepts encountered in 36 lines per sample, with ten random fields per sample observed under a microscope at ×100 magnification^21,22^. DI was determined by dividing the number of destroyed alveoli by the total number of alveoli counted, with an average of 5 different sections examined per sample under a microscope at ×100 magnification. Alveolar destruction was defined by criteria such as at least two alveolar wall defects, at least two intraluminal parenchymal rags in alveolar ducts, clearly abnormal morphology, or classic emphysematous changes in the lung^23^.

TUNEL staining was conducted to evaluate apoptosis levels in lung tissue using an in-situ apoptosis detection kit (Shanghai Yisheng Biotech, China). The apoptotic index (AI) was calculated in lung tissue from each subject to assess lung parenchyma apoptosis. AI was determined as the percentage of TUNEL-positive nuclei out of over 3000 randomly selected nuclei at ×400 magnification. Fields containing non-parenchymal structures, such as large airways or vessels, were excluded^4^.

Apoptotic cells were detected using an annexin V-fluorescein isothiocyanate (FITC)/propidium iodide (PI) cell apoptosis kit (KeyGEN BioTECH, China) following the manufacturer's instructions. Briefly, cells were washed and incubated with 500 μL of 1× binding buffer containing 5 μL of annexin V-FITC and 5 μL of PI for 15 minutes in the dark. Apoptosis was assessed by flow cytometry (BD Biosciences), with early apoptosis determining the percentage of apoptotic cells. Each experiment was conducted in triplicate.

### Real-time RT-PCR

MLE-12 cells were exposed to 5% cigarette smoke extract (CSE) for 24 hours. RNA was isolated using TRIzon reagent (Cwbio, China) following the manufacturer's protocol. Reverse transcription of the first-strand cDNA was carried out using the RevertAid First Strand cDNA Synthesis Kit (Thermo Fisher Scientific, USA). Real-time quantitative PCR was conducted using the All-in-OneTM Qpcr Mix (GeneCopoeiaTM) on a CFX96™ PCR machine (Bio-Rad, Hercules, CA, USA). All procedures were performed according to the manufacturer's instructions, with β-actin used as the internal control. The comparative C(T) method was employed for realtime PCR data analysis, and mRNA expression levels were normalized to β-actin^24^.

### Western blotting

MLE-12 cells were treated with 5% cigarette smoke extract (CSE) for 24 hours. Following treatment, cells were harvested in RIPA cell lysis buffer supplemented with protease inhibitors (Merck, Germany), and protein concentrations were quantified using the BCA protein assay. Subsequently, 20 μg of protein extracts were separated by SDS-PAGE using 12% and 8% polyacrylamide gels, followed by transfer to polyvinylidene difluoride (PVDF) membranes. The membranes were then blocked with 1× TBST containing 5% skim milk and incubated overnight at 4°C with primary antibodies against Shh, Gli1, Ptch1, Bcl-2, Caspase3, and β-actin (sourced from Proteintech, USA, and Abcam, UK). Following primary antibody incubation, the membranes were probed with a horseradish peroxidase-conjugated goat anti-rabbit IgG antibody (Proteintech, USA) for 1.5 hours at room temperature. Immunoreactivity was visualized using an enhanced chemiluminescence kit according to the manufacturer's instructions. Protein expression levels were normalized to β-actin expression.

### Statistical analysis

The data analysis utilized Statistical Package for Social Sciences (SPSS) version 21.0 and R software version 3.6.2 (R Foundation for Statistical Computing). Descriptive statistics were reported as means with standard deviation (SD). One-way ANOVA and Kruskal-Wallis tests were conducted to analyze the data from each group. Statistical significance was defined as p < 0.05.

## RESULTS

### Expression of MiR-210 is down-regulated explicitly in CSE-induced MLE-12

**Figure 1:**
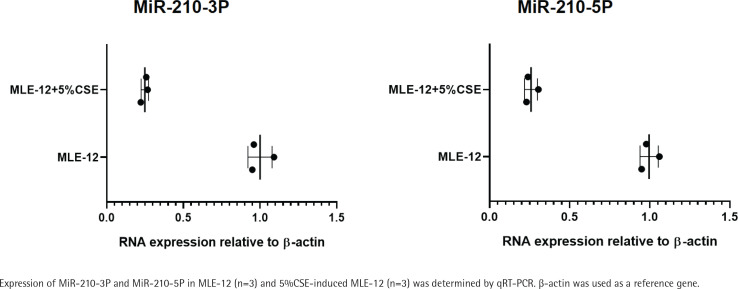
Expression of MiR-210 is specifically down-regulated in CSE-induced MLE-12

First, we detected the expression of MiR-210 in MLE-12 and 5% CSE-induced MLE-12. Contrary to our assumptions, MiR-210, including MiR-210-3p and MiR-210-5p, was the most down-regulated MicroRNA in CSE-induced MLE-12 compared with MLE-12 (Figure 1). These results indicate that MiR-210 may have an important role in COPD.

### Effect of CSE on the expression of Shh pathway and apoptosis in MLE-12

**Figure 2:**
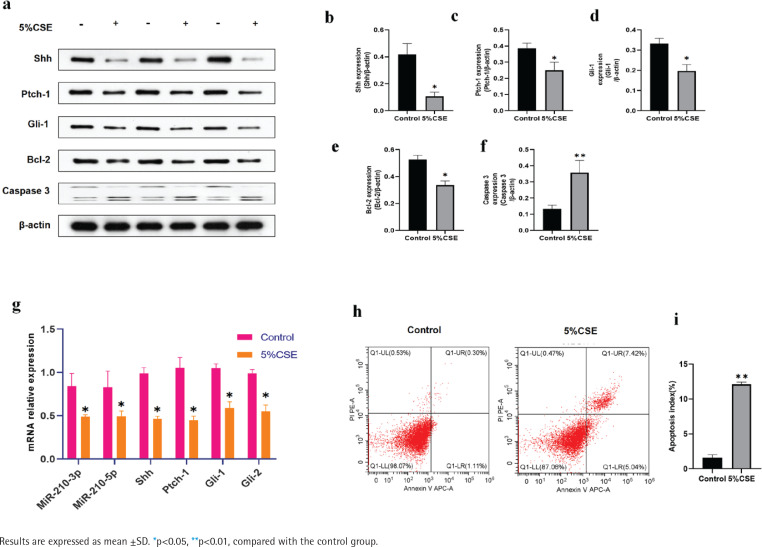
Effect of CSE on expression of Shh pathway and apoptosis in MLE-12: a) Immunoblotting was conducted using MLE-12 from the control and 5%CSE groups; b–f) The relative expressions of Shh, Ptch-1, Gli-1, Bcl-2, and Caspase 3 in MLE-12; g) Expression of MiR-210-3P, MiR-210-5P, Shh, Ptch-1, Gli-1 and Gli-2 were measured by qRT-PCR from the control and 5%CSE groups; h) Flow cytometry was conducted in MLE-12 from the control and 5%CSE groups; i) Statistical analysis of the AI in two groups.

MLE 12 was treated with 5% CSE for 24 h before Western blotting. The same was done for the detection of apoptosis and mRNA expression of the Shh pathway. After CSE treatment, the protein expression of the Shh pathway (Shh, Ptch1, Gli1) decreased, and so did the anti-apoptotic factor Bcl-2, while the level of the apoptotic factor Caspase 3 was significantly increased (Figures 2a–2f). Furthermore, qRT-PCR showed that there were lower MiR-210, Shh, Ptch1, Gli1 and Gli2 mRNA levels in the CSE group than in control group subjects (Figure 2g). Following CSE treatment, a notable elevation in apoptosis was observed in the 5% CSE group compared to the control group (Figures 2h and 2i). These findings suggest a potential inhibition of the Shh pathway in MLE-12 cells by CSE, leading to enhanced apoptosis.

### MiR-210 attenuates CSE-induced apoptosis via the Shh pathway in MLE-12

We aimed to investigate whether MiR-210 could mitigate apoptosis in MLE-12 cells exposed to CSE via the Shh pathway. Immunoblotting analysis revealed decreased levels of Shh, Ptch1, Gli1, and Bcl-2 proteins, along with increased levels of Caspase 3 protein in the CSE group compared to the control group. Moreover, compared to the CSE + Vector group, the Si-MiR-210 group exhibited significant reductions in Shh, Ptch1, Gli1, and Bcl-2 protein levels, alongside a notable increase in Caspase 3 protein level (Supplementary file Figures 1a–1f). Additionally, qRT-PCR analysis showed decreased levels of MiR-210, Shh, Ptch1, and Gli1 mRNA in the CSE group compared to the control group. Similarly, compared to the CSE + Vector group, the Si-MiR-210 group demonstrated significantly reduced mRNA levels of MiR-210, Shh, Ptch1, and Gli1 (Supplementary file Figures 1g–1j). Next, we performed flow cytometry to clarify whether decreased MiR-210 was associated with apoptosis in CSE-exposed MLE 12. The results demonstrated significantly fewer apoptotic cells in the control and CSE + Vector groups than in the CSE and Si-MiR-210 groups (Supplementary file Figures 1k–1l). These results indicate that decreased MiR210 could facilitate CSE-induced apoptosis in MLE 12. Considering the simultaneously decreased Shh expression, it is possible to assume that the down-regulated MiR-210 level leads to the decreased Shh pathway expression in CSE-exposed MLE 12.

### MiR-210 attenuates CS-induced apoptosis via the Shh pathway in the mouse model

Due to the decreased MiR-210 mRNA levels observed in CSE-exposed MLE-12 cells, we investigated whether altering MiR-210 mRNA levels or activity could mitigate emphysema and pulmonary apoptosis in mouse models. Mice were intratracheally depleted of MiR-210 using a lentiviral delivery system (10^7^ plaque-forming units per mouse) and subsequently exposed to cigarette smoke (CS). In CS-exposed mice, both the mean linear intercept (MLI) and destruction index (DI) values significantly increased compared to the control group, whereas they decreased in the CSE + Vector group compared to the Si-MiR-210 group. Additionally, Si-MiR-210-treated mice exhibited emphysematous changes with exacerbated MLI and DI (Supplementary file Figures 2a and 2b). Immunoblotting further revealed higher Caspase 3 levels and lower levels of Shh, Ptch1, Gli1, and Bcl-2 proteins in the Si-MiR-210 group compared to both the CS + Vector and control group subjects (Supplementary file Figures 2c–2h).

Notably, qRT-PCR showed that the mRNA levels of MiR-210, Shh, Ptch1, and Gli1were significantly decreased in the Si-MiR-210 group compared with the CS + Vector and control groups (Supplementary file Figures 2i–2l). TUNEL staining also showed more pulmonary apoptosis in the Si-MiR-210 group than in the CS + Vector and control groups (Supplementary file Figures 2m and 2n). These results indicate that MiR-210 gene silencing aggravated emphysema and pulmonary apoptosis and decreased Shh pathway expression in CS-exposed mice.

## DISCUSSION

Our results showed that MiR-210 mRNA levels were significantly down-regulated in the CSE-induced MLE 12, and MiR-210 down-expression can facilitate CSE-induced cell apoptosis and emphysema via the Shh pathway in mice. *In vitro*, MiR-210 gene silencing can aggravate the apoptosis of CSE-exposed MLE 12. Moreover, MiR-210 regulated the Shh pathway and promoted its expression. These findings illustrate that MiR-210 is involved in cigarette smoke extract-induced apoptosis of MLE-12 via the Shh signaling pathway in COPD.

MiRNAs play pivotal roles as key regulators of transcription and translation, orchestrating intricate interactions with DNAs, RNAs, and proteins over recent decades^25,26^. Notably, MiR-210 stands out as a significant player in numerous tumor development processes, characterized by its elevated expression in various cancers^27^. It is closely linked to tumor progression, including advanced stages and widespread metastasis. MiR-210 targets a diverse array of genes involved in pivotal cellular processes such as mitochondrial metabolism, angiogenesis, DNA repair, and cell survival, underscoring its multifaceted impact on cancer biology^18^. We discovered, quite interestingly, that MiR-210 acted as a crucial regulator in COPD development, attenuating MLE 12 apoptosis and promoting Shh signaling pathway expression *in vitro* and *in vivo*. Devlin et al.^18^ identified MiR-210 as the sole miRNA consistently upregulated in both normal and transformed hypoxic cells across various published studies. Its expression level may serve as a reflection of hypoxia-inducible factor activity, even in circumstances of normoxic activation of this transcription factor^28,29^. Additionally, Juan et al. conducted a study utilizing 28 clear-cell type human renal cell carcinoma (ccRCC) samples obtained from patient-matched specimens. Through highthroughput quantitative real-time polymerase chain reaction analysis of microRNA expression levels, they discovered that MiR-210, induced by hypoxia, was significantly overexpressed in ccRCC^30^. Likewise, another investigation showed that the upregulation of hsa-miR-210 was triggered by hypoxia in a manner reliant on HIF-1alpha and VHL. Additionally, the levels of hsa-miR-210 expression in breast cancer specimens were recognized as an independent prognostic indicator^31^. However, MiR-210, including MiR-210-3p and MiR-210-5p, was the most down-regulated MiRNA in CSE-induced MLE-12 compared with MLE-12 in our study. Recent evidence has highlighted the mechanisms through which MiR-210 targets genes in bone marrow-derived mesenchymal stem cells. Using qRT-PCR arrays for rat apoptotic genes, computational target gene analyses, and luciferase reporter assays, researchers identified FLICE-associated huge protein (FLASH)/caspase-8-associated protein-2 (Casp8ap2) as a target gene of MiR-210. MiR-210 directly antagonized an apoptotic component, Casp8ap2, and the relevance of this for cancer is currently unknown^32^. What we found, consistent with recent evidence, was that MiR-210 directly antagonized an apoptotic component, Caspase 3, presumably through regulation of Shh signaling pathway.

We also investigated the anti-apoptotic effect of the Shh signaling pathway in the CSE-induced apoptosis of MLE 12. After CSE treatment, the protein and mRNA expression of the Shh pathway decreased, as did the anti-apoptotic factor Bcl-2, while the level of the apoptotic factor Caspase 3 was significantly increased. Wang et al.^33^ found that itraconazole treatment inhibited hedgehog pathway key molecular expression, such as Shh and Gli1, resulting in the promotion of apoptosis and autophagy in breast cancer. Another study also advanced our knowledge that polydatin can protect kidneys against ischemia/ reperfusion injury by enhancing antioxidant capacity and decreasing cell apoptosis by activating the Shh signaling pathway^12^. Research on the anti-apoptotic effects of the Shh pathway in pulmonary diseases is relatively sparse. Our findings indicate that the Shh pathway may play a role in reducing CSE-induced apoptosis in MLE-12 cells. Shh protein appears to promote cell proliferation, thereby competing with apoptotic processes. Previous studies have mainly examined the Shh pathway in the context of embryonic lung development, suggesting it promotes stem cell proliferation^34^. Overexpression of Shh protein has been shown to assist in lung injury repair by increasing lung stem cells. Our study's results are consistent with these earlier findings. Based on the existing evidence and our current data, we propose that the Shh pathway is involved in regulating CSEinduced apoptosis in MLE-12 cells.

### Limitations

Our study has several limitations. First, the small sample size may not fully validate the accuracy of the findings. Secondly, we did not thoroughly investigate the genes involved in the MiR-210/Shh signaling pathway axis. Further research is required to understand how MiR-210 inhibition contributes to COPD progression. Thirdly, previous studies indicated that both epithelial and endothelial cells are implicated in emphysema. The interactions between these cell types are complex and need further exploration.

## CONCLUSIONS

Our results suggest that MiRNA-210 is involved in cigarette smoke extract-induced apoptosis of MLE-12 via the Shh signaling pathway. The present study reveals that MiRNA-210 may be a key regulator of cellular apoptosis and could be explored as a potential therapeutic target in the future.

## Supplementary Material



## Data Availability

The data supporting this research are available from the authors on reasonable request.
