# Supplementary material for: MiRNA-210 is involved in cigarette smoke extract-induced apoptosis of MLE-12 via the Shh signaling pathway
Source: Tob Induc Dis. 2024 May 29;22:10.18332/tid/186643. doi: 10.18332/tid/186643 (PMC11135024; doi:10.18332/tid/186643)
Supplement: Supplementary file 1 [file TID-22-92-s1.pdf]

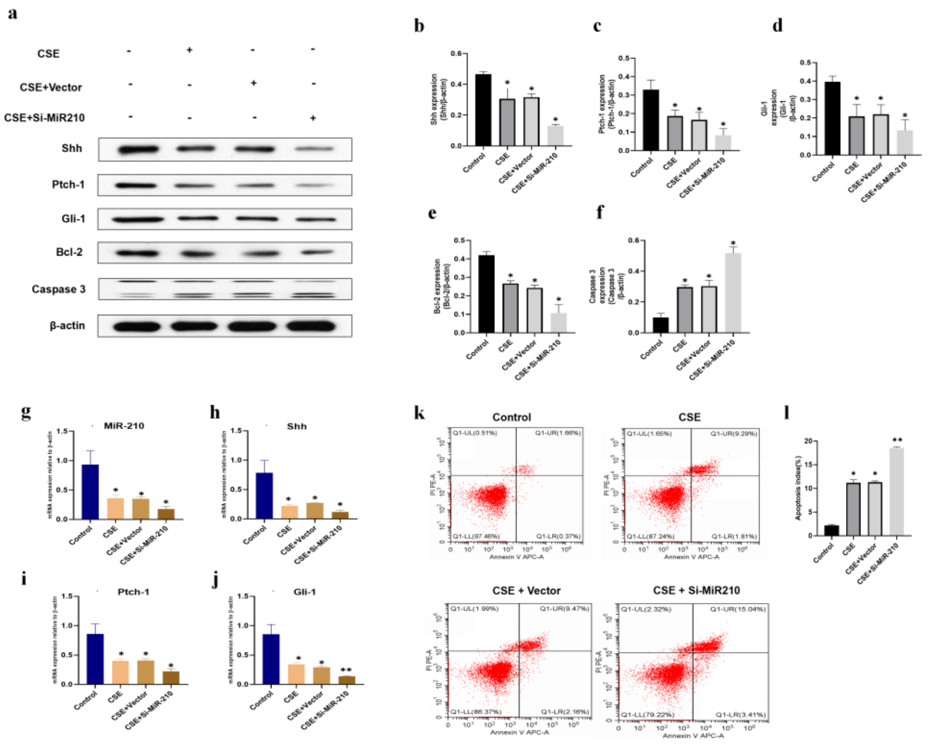

Figure 1 MiR-210 attenuates CSE-induced apoptosis via Shh pathway in MLE-12

a Immunoblotting was conducted using MLE-12 from the control, CSE, CSE + Vector and CSE + Si-MiR210 groups. (b-f) The relative expressions of Shh, Ptch-1, Gli-1, Bcl-2, and Caspase 3 in MLE-12. Results are expressed as mean±SD. *P<0.05 compared with the control group. (b-j) Expression of MiR-210, Shh, Ptch-1, and Gli-1 were measured by qRT-PCR from the control, CSE, CSE + Vector and CSE + Si-MiR210 groups.*P<0.05 compared with the control group. k Flow cytometry was conducted in MLE-12 from the control, CSE, CSE + Vector and CSE + Si-MiR210 groups. l Statistical analysis of the AI in different groups. Results are expressed as mean±SD.*P<0.05.** P < 0.01.

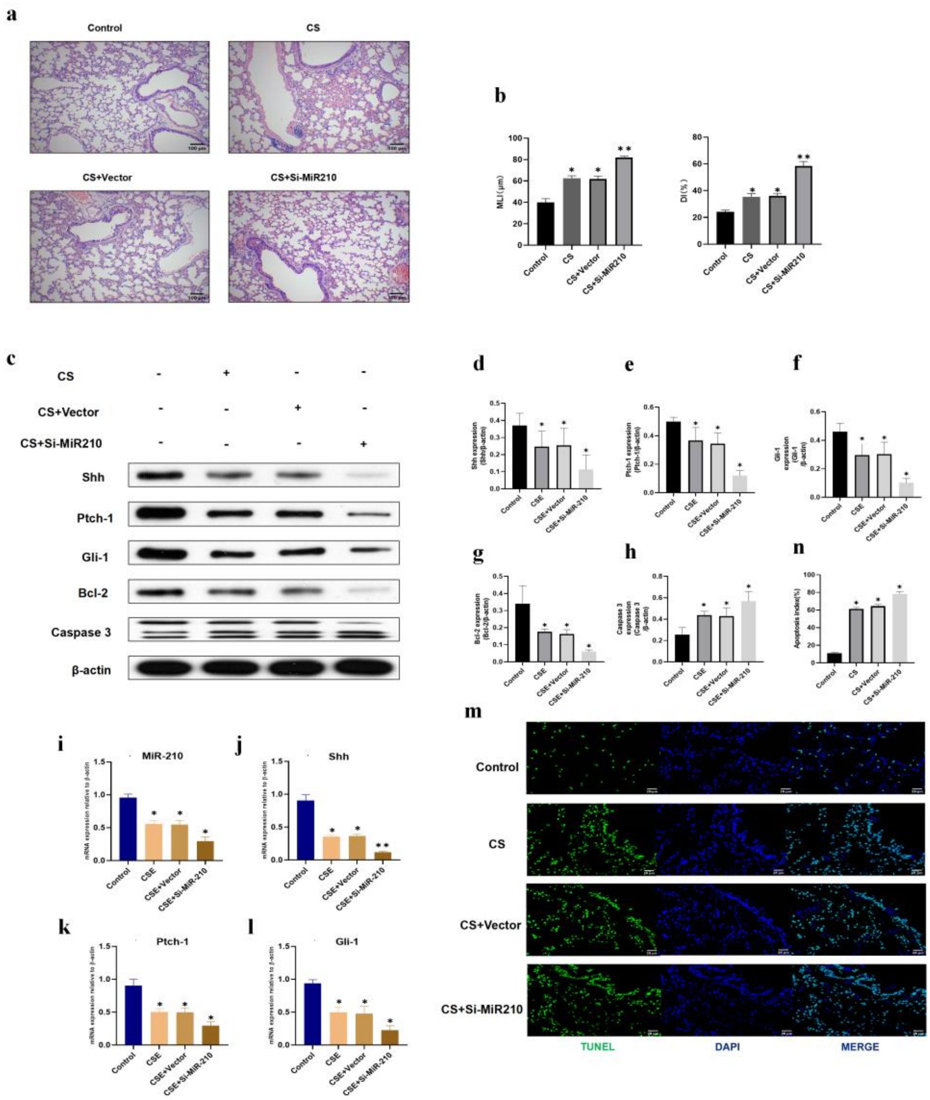

Figure 2 MiR-210 attenuates CS-induced apoptosis via the Shh pathway in the mouse model
a HE staining of lungs from the control, CS, CS + Vector and CS + Si-MiR210 groups. Scale bar = 100 μm. b Morphometric measurements of MLI (μm) and DI (%) were performed in each group. Results are expressed as mean±SD. *P<0.05 compared with the control group. c Immunoblotting was conducted using MLE-12 from the control, CS, CS + Vector and CS + Si-MiR210 groups. (b-f) The relative expressions of Shh, Ptch-1, Gli-1, Bcl-2, and Caspase 3 in MLE-12. Results are expressed as mean±SD. *P<0.05 compared with the control group. (i-l) Expression of MiR-210, Shh, Ptch-1, and Gli-1 were measured by qRT-PCR from the control, CS, CS + Vector and CSE + Si-MiR210 groups.*P<0.05 compared with the control group. m TUNEL staining was conducted in MLE-12 from the control, CS, CS + Vector and CS + Si-MiR210 groups. n Statistical analysis of the AI in different groups. Results are expressed as mean±SD.*P<0.05.** P < 0.01.

©2024 Dai Z. et al.
